# Effect of Tai Chi Exercise on Fatigue in Renal Transplant Recipients: A Randomized Controlled Trial

**DOI:** 10.1155/nrp/2584203

**Published:** 2026-10-13

**Authors:** Siyu Qiao, Chunting Liu, Yuxi Lu, Hongqin Li, Wenwen Han

**Affiliations:** ^1^ School of Nursing Jilin University Changchun Jilin China jlu.edu.cn; ^2^ Department II of Urology—Organ Transplantation Center The First Hospital of Jilin University Changchun Jilin China jlu.edu.cn

**Keywords:** exercise, fatigue, kidney, Tai Chi, transplants

## Abstract

**Background:**

Fatigue is a prevalent symptom in kidney transplant patients that not only affects quality of life but may also impact long‐term graft survival. While Tai Chi has been shown to effectively reduce fatigue in patients with chronic diseases, research in kidney transplant recipients remains limited.

**Objective:**

To explore the effects of Tai Chi exercise on fatigue among kidney transplant recipients and to investigate the effectiveness and safety of Tai Chi‐based rehabilitation.

**Methods:**

A prospective, single‐center, parallel‐arm randomized controlled trial was conducted in Northeast China. The study period for patient recruitment and intervention implementation was from 1 June 2025, to 12 October 2025. Eligible participants were kidney transplant recipients aged 18–60 years who were at least 1 month post‐transplantation. Sixty‐four kidney transplant patients were randomly allocated (1:1) to the intervention group (Tai Chi exercise) or the control group (routine care only). The intervention group received a 12‐week Tai Chi exercise program. The primary outcome was fatigue severity assessed at 12 weeks.

**Results:**

After 12 weeks of Tai Chi intervention, there were statistically significant differences in total fatigue scores between the two groups of renal transplant recipients. Sleep quality and 6‐min walk distance were significantly improved.

**Conclusions:**

Tai Chi exercise is a gentle and well‐tolerated exercise intervention that can effectively improve fatigue in renal transplant recipients.

**Trial Registration:** International Traditional Medicine Clinical Trial Registry: 2026000360.

**Summary**
• Implication for the Professional and/or Patient Care
◦ Tai Chi exercise provides a scientifically grounded and safe exercise intervention for managing fatigue symptoms in renal transplant recipients.
• Impact
◦ This study showed that Tai Chi intervention can effectively improve fatigue in renal transplant recipients.
• Reporting Method
◦ We adhered to the CONSORT checklist for reporting results.
• Patient or Public Contribution
◦ No patients or members of the public participated in the study design, data analysis, or interpretation.
• What is already known
◦ Fatigue represents a prevalent and burdensome symptom among kidney transplant recipients, substantially impacting daily functioning, occupational performance, and quality of life.
◦ Tai Chi has been shown to enhance both physical and mental well‐being and quality of life, while also yielding positive effects on fatigue reduction.
◦ However, due to fear of compromising graft function, the majority of kidney transplant recipients are either sedentary or engage in very limited physical activity.
• What this paper adds
◦ A nurse‐led three‐phase Tai Chi exercise prescription can effectively improve fatigue severity, daily activity capacity, and quality of life among kidney transplant recipients.
◦ Kidney transplant recipients showed high adherence to the Tai Chi exercise intervention. As a traditional Chinese exercise featuring slow‐paced rhythm and gentle movements, Tai Chi is more readily accepted by patients.
• Implications for policy/practice
◦ As a low‐intensity, slow‐paced aerobic exercise, Tai Chi demonstrates an excellent safety profile and is particularly suitable for home‐ or community‐based settings. It effectively alleviates transplant recipients’ common concerns about exercise and knowledge deficits, providing patients with a clear, evidence‐based, and safe exercise prescription.

## 1. Introduction

Epidemiological data indicate that approximately 850 million people worldwide suffer from chronic kidney disease (CKD) [1], with China having the largest CKD patient population [2]. When CKD progresses to end‐stage renal disease (ESRD), kidney transplantation is recognized as one of the most effective therapeutic modalities [3]. With the continuous advancement of kidney transplantation technology, long‐term graft survival rates have been significantly improved [4]. Simultaneously, the health‐related quality of life of kidney transplant recipients has been substantially enhanced [5]. Although kidney transplantation typically yields better survival, quality of life, and overall functional recovery than long‐term dialysis, the postoperative functional trajectories of transplant recipients vary considerably, with some achieving near‐normal function while others endure persistent functional deficits. A constellation of factors—including surgical trauma, prolonged immunosuppressive therapy and corticosteroid administration, frequent post‐transplantation monitoring, and transplant‐related complications—may precipitate various adverse symptoms in kidney transplant recipients, including fatigue [6], tremor [7], emotional fluctuations and anxiety [8], as well as cognitive impairment [9]. Among these manifestations, fatigue emerges as a prevalent yet frequently underappreciated symptom. The reported incidence of fatigue in kidney transplant recipients ranges from 40% to 83.48% [10–12]. Fatigue not only substantially reduces patients’ health‐related quality of life [13], but also demonstrates complex associations with sleep disorders and affective disturbances, forming a multidimensional symptom cluster [14] that exacerbates psychological morbidity. Of particular clinical significance, fatigue may undermine treatment adherence [15], thereby compromising long‐term allograft outcomes [16].

Exercise interventions are effective in alleviating fatigue symptoms across multiple populations and conditions [17–19]. However, there are relatively few exercise intervention studies targeting fatigue in kidney transplant recipients. A randomized controlled trial has substantiated that exercise‐based interventions, either as standalone programs or in combination with dietary modification, significantly attenuate fatigue symptoms and decrease the occurrence of severe fatigue in kidney transplant recipients [20]. A 3‐month supervised physical exercise intervention program significantly ameliorates fatigue symptoms in kidney transplant recipients [21]. Graded aerobic exercise combined with resistance training has also been demonstrated to effectively improve functional capacity, muscle strength, and fatigue severity in kidney transplant recipients [22]. However, despite the established benefits of exercise interventions, most kidney transplant recipients remain largely sedentary or engage in minimal physical activity. This lack of engagement is multifactorial, driven by physiological and psychological mechanisms. Long‐term corticosteroid therapy causes muscle loss and osteoporosis, which impair exercise capacity [23]. Side effects of immunosuppressive agents—including muscular atrophy and metabolic syndrome—further reduce exercise tolerance [21]. Beyond treatment‐related effects, obesity and peripheral vascular disease are independent comorbidity‐level risk factors for physical inactivity [24]. Psychological factors also contribute, notably kinesiophobia—the fear that exercise may damage the transplanted kidney—which has been suggested as a barrier to physical activity [25]. These barriers ultimately translate into physical inactivity, which is closely intertwined with fatigue [21]. The relationship is reciprocal: inactivity exacerbates fatigue, while fatigue in turn discourages exercise, trapping patients in a vicious cycle of progressive functional decline and worsening symptom burden [26].

Among the array of exercise modalities currently employed in clinical practice, Tai Chi has emerged as a particularly promising intervention owing to its unique holistic psychosomatic benefits. As a traditional Chinese medicine‐based exercise intervention, Tai Chi synthesizes gentle physical movements, controlled breathing techniques, and mindfulness meditation, thereby conferring some advantages, including physiologically moderate exercise intensity, relative safety, accessibility for novice practitioners, and long‐term adherence potential. Accumulating empirical evidence has substantiated the efficacy of Tai Chi in significantly ameliorating fatigue symptomatology across heterogeneous populations afflicted with chronic diseases [27, 28]. A rigorously designed randomized controlled trial in breast cancer patients demonstrated that Tai Chi intervention effectively mitigates the integrated fatigue‐sleep disturbance‐depression symptom cluster [29]. Prior research has further demonstrated that Tai Chi exercise improves health‐related quality of life and physical function in patients with ESRD; however, fatigue has not been specifically examined as an outcome in this population [30]. Together, these lines of evidence support the rationale for evaluating the potential benefits of Tai Chi on fatigue in kidney transplant recipients. Despite the growing evidence for exercise‐based fatigue management, research in kidney transplant recipients remains limited. Therefore, the objectives of this research are to determine the effectiveness of Tai Chi exercise in improving fatigue symptoms among kidney transplant recipients. We hypothesized that participants assigned to the Tai Chi group would achieve significantly greater improvements in fatigue symptoms compared with those in the control group.

## 2. Method

### 2.1. Study Design and Setting

This was a single‐center, two‐arm, parallel‐group, randomized controlled trial. The primary objective was to investigate the efficacy of Tai Chi exercise as an intervention for fatigue in kidney transplant recipients. We conducted this study at a tertiary hospital in Jilin Province, China. Patient recruitment and intervention implementation spanned from 1 June 2025, to 12 October 2025. Ethical approval was obtained from the institutional ethics committee prior to the study (reference number: 25K128‐001).

### 2.2. Participants

Participants were recruited at the Renal Transplant Follow‐up Clinic of a tertiary hospital in Jilin Province from June 9, 2025, to June 30, 2025.

The inclusion criteria were as follows: (1) time since kidney transplantation ≥ 1 month; (2) age 18–60 years; (3) stable transplant renal function (serum creatinine < 200 μmol/L, glomerular filtration rate > 30 mL/min/1.73 m^2^); (4) ability to move freely and perform limb exercises; and (5) capacity to complete questionnaires, voluntary participation throughout the study, and signed informed consent.

The exclusion criteria were as follows: (1) presence of language, hearing, or comprehension impairment, or serious mental illness; (2) patients with severe complications; (3) comorbidities with severe cardiac, hepatic, cerebral, or pulmonary disease; (4) patients engaged in regular structured exercise programs; and (5) current participation in other clinical trials that may affect the outcomes of this study.

The withdrawal criteria were as follows: (1) patients with disease deterioration or adverse events (such as transplant kidney rejection, graft failure, return to dialysis, etc.), exercise intervention will be discontinued based on physician judgment; (2) non‐adherence to the intervention protocol; and (3) withdrawal request from the participant or family members.

### 2.3. Study Procedures

The researcher implemented a standardized Tai Chi intervention protocol for participants in the experimental group. The principal investigator completed formal training in Tai Chi at the Tai Chi Club of a tertiary hospital in Jilin Province until demonstrating complete proficiency in the techniques. Two trained nurses recruited eligible participants at the kidney transplant follow‐up outpatient clinic. All participants were required to provide written informed consent prior to enrollment. Baseline data were collected from all consenting participants, following which eligible participants were randomly allocated to either the intervention or control group using a computerized randomization system. Both groups received standard care, and the patients in the intervention group were taught Tai Chi exercise by the researcher. Following the 12‐week intervention period, one designated nurse who was blinded to group allocation collected postintervention data from all participants to assess study outcomes.

### 2.4. Sample Size Calculation

Sample size was calculated based on published literature data for the physical fatigue dimension of the Multidimensional Fatigue Inventory (MFI) [21]. Using mean scores of 6.23 (SD = 1.34) for the intervention group and 10.34 (SD = 2.20) for the control group as reference values, with a significance level of *α* = 0.05, a statistical power of 1 − *β* = 0.9, and a 1:1 allocation ratio, superiority testing for quantitative data was performed using PASS 15 software. The calculated sample size was 26 participants per group. Accounting for an anticipated dropout rate of 20%, 32 participants were required in each group, resulting in a total sample size of 64 participants.

### 2.5. Randomization, Concealment, and Blinding

A simple randomization method was employed to allocate participants in a 1:1 ratio to the intervention and control groups. Sixty‐four participants were sequentially numbered from 1 to 64, with each number representing an individual subject. Using Excel software, a randomization sequence containing 64 random numbers was generated and sorted by magnitude. The first 32 participants, corresponding to the lower random numbers, were allocated to the intervention group, while the remaining 32 participants were allocated to the control group. To conceal allocation, each random number was placed in a sealed, opaque envelope and distributed to participants by an independent research staff member not involved in participant recruitment or outcome assessment. Both outcome assessors and data analysts were blinded to group allocation throughout the study period to minimize potential bias in outcome measurement and statistical analysis.

### 2.6. Interventions

#### 2.6.1. Intervention: Tai Chi Exercise

##### 2.6.1.1. Development of the Tai Chi Intervention Protocol

After a comprehensive comparison, the present study selected the gentler Yang‐style Tai Chi, which is characterized by relatively soft force exertion, and further established the 24‐form simplified Tai Chi as the specific exercise modality, given its widespread clinical application and ease of learning [31]. This form features a rich variety of postures, emphasizes the coordination between the upper and lower limbs, and involves no excessive or forceful movements, thereby offering a high safety profile and rendering it well‐suited for the postoperative rehabilitation of kidney transplant recipients. The intervention protocol was developed based on the FITT principle, which encompasses four core elements: frequency, intensity, time, and type. Based on a comprehensive literature review and internal group discussions, the research team modified the exercise content by integrating clinical guidelines for renal transplant recipients and the team’s clinical expertise. Consequently, four additional essential elements were incorporated into the original FITT framework, resulting in an eight‐component preliminary Tai Chi protocol: exercise assessment, exercise phase, exercise mode, frequency, intensity, duration, intervention format, and quality control. To further refine the protocol, a two‐round Delphi expert consultation was conducted with 12 experts, including clinical specialists in renal transplantation (medical and nursing), certified Tai Chi rehabilitation experts, and research methodologists. The expert authority coefficient (Cr) was 0.88. Following the consultation process, the final version of the Tai Chi intervention protocol was established.

##### 2.6.1.2. Description of the Tai Chi Intervention Protocol

In addition to standard exercise counseling, participants in the intervention group received a structured Tai Chi exercise prescription implemented in three sequential phases over 12 weeks. Phase 1 (Weeks 1–2: training) involved hospital‐based systematic instruction of 24‐form Yang‐style Tai Chi (30 min/session, 3 sessions/week, low‐to‐moderate intensity), supplemented by educational materials via a dedicated WeChat group. Phase 2 (Weeks 3–6: guided practice) consisted of supervised sessions three times weekly at moderate intensity, with technical refinement provided in person or via Tencent Meeting. Participants progressed to Phase 3 (Weeks 7–12: independent practice) after passing an instructor‐led evaluation, during which they performed self‐directed practice five times weekly for 30 min at moderate intensity. The details of the intervention are shown in Table 1.

**TABLE 1:** Details of the Tai Chi intervention protocol.

Item	Primary category	Details
1. Exercise assessment	1.1 Pre‐intervention medical evaluation	Prior to Tai Chi intervention initiation, qualified physicians evaluated contraindications (e.g., acute rejection episodes, uncontrolled severe complications, severe physical debility) and assessed exercise intensity and range of motion tolerance.
	1.2 Baseline physical assessment	A 6‐min walk test (6MWT), grip strength assessment, and simple balance evaluation were performed by physicians and transplant nurses to evaluate exercise tolerance, upper limb muscle strength, and balance capacity.

2. Intervention phases	2.1 Overall structure	The 12‐week intervention was divided into three sequential phases following the principle of progressive advancement: intensive training, one‐on‐one guided practice, and independent practice.
	2.2 Phase 1: Intensive training (Weeks 1–2)	Certified instructors provided systematic instruction in 24‐form Yang‐style Tai Chi and warm‐up/cool‐down exercises. The objective was to enable participants to master foundational techniques.
	2.3 Phase 2: One‐on‐one guided practice (Weeks 3–6)	Instructors provided individualized technical correction and refinement. The objective was to develop proficiency in standardized movements and independent practice capability.
	2.4 Phase 3: Independent practice (Weeks 7–12)	Following instructor‐led competency assessment, participants performed self‐directed practice. Individual technical optimization continued during follow‐up visits.

3. Exercise mode	3.1 Exercise structure	Each session comprised three components: warm‐up exercise + Tai Chi practice + cool‐down exercise.
	3.2 Tai Chi form	The 24‐form Yang‐style Tai Chi (simplified form), officially developed in 1956 by the State General Administration of Sports, features smooth and gradual movements.
	3.3 Warm‐up and cool‐down	Upper and lower limb stretching exercises combined with slow walking (5–10 min each).

4. Exercise frequency	4.1 Progressive principle	Exercise frequency increased progressively across intervention phases.
	4.2 Phases 1–2 (Weeks 1–6)	3 training sessions per week, one session per day.
	4.3 Phase 3 (Weeks 7–12)	5 training sessions per week, one session per day.

5. Exercise intensity	5.1 Progressive principle	Exercise intensity increased progressively across intervention phases.
	5.2 Phase 1: Intensive Training	Low‐intensity exercise (50%–60% of maximum heart rate).
	5.3 Phase 2: Guided practice	Low‐to‐moderate intensity exercise (50%–85% of maximum heart rate).
	5.4 Phase 3: Independent practice	Moderate‐intensity exercise (60%–85% of maximum heart rate).

6. Exercise duration	6.1 Session duration	Each session duration remained constant at 30–45 min across all phases.
	6.2 Component duration	Warm‐up: 5–10 min; Tai Chi practice: 20 min; cool‐down: 5–10 min.

7. Intervention format	7.1 Phases 1–2 (Weeks 1–6)	In‐person, hospital‐based intervention. Participants were required to attend supervised sessions at the hospital.
	7.2 Phase 3 (Weeks 7–12)	Hybrid format combining in‐person and home‐based practice. Participants could choose hospital‐based or home‐based practice with online guidance.

8. Quality control	8.1 Professional instruction	Certified Tai Chi instructors provided systematic training and real‐time technical guidance throughout all intervention phases.
	8.2 WeChat support group	A dedicated WeChat group was established for teaching resources, instructor feedback, and video submission. Participants unable to attend in‐person sessions submitted practice videos for review. Independent‐phase home‐practice participants submitted weekly practice videos.
	8.3 Exercise log documentation	Standardized exercise logs were provided to document training duration, maximum heart rate, subjective exercise experience, and difficulties encountered. Logs were collected and organized in three‐line table format. Adherence was monitored and promoted throughout Phase 3.

#### 2.6.2. Control: Standard Care

Standard exercise counseling was provided to all participants regarding appropriate physical activity based on individual clinical status. Kidney transplant recipients were advised to engage in graduated physical activity commensurate with their current functional capacity and overall health status. As recipients’ physical condition improved during the recovery period, the intensity and volume of activity were progressively increased in accordance with individual tolerance and clinical assessment. The exercise prescription encompassed routine daily activities performed postdischarge, including moderate‐intensity walking and aerobic exercise modalities.

### 2.7. Outcome Measures

The primary outcome measure was fatigue severity, assessed using the Multidimensional Fatigue Inventory (MFI‐20). Secondary outcome measures included quality of life, anxiety and depression, sleep quality, 6‐min walk distance (6MWD), body mass index (BMI), and serum creatinine. Measurements were conducted at baseline and at the end of the 12‐week intervention period.

#### 2.7.1. Personal Information Questionnaire

Demographic and clinical information were collected using a self‐administered questionnaire. The questionnaire included demographic variables (gender, age, marital status, and educational level) and uremia‐related clinical variables (primary renal disease, duration of illness, dialysis modality, and duration of dialysis treatment).

#### 2.7.2. Fatigue

Fatigue severity was assessed using the 20‐item self‐reported MFI‐20, which was culturally adapted and validated in Chinese populations [32]. A 5‐point Likert scale was employed, with individual item scores ranging from 1 to 5. Total scores ranged from 20 to 100, with higher scores indicating greater severity of fatigue. The use of the MFI‐20 in this study was officially approved by the original scale authors.

#### 2.7.3. Quality of Life

Health‐related quality of life was assessed using the 36‐Item Short‐Form Health Survey (SF‐36), which has been well validated among Chinese populations [33]. The SF‐36 comprises eight health domains: Physical Functioning, Role‐Physical, Bodily Pain, General Health, vitality, Social Functioning, Role‐Emotional, and Mental Health. Permission for the use of this scale was granted by the original authors.

#### 2.7.4. Anxiety and Depression

Anxiety and depression symptoms were assessed using the Hospital Anxiety and Depression Scale (HADS), a 14‐item self‐report questionnaire [34]. Seven items assess depressive symptoms, and seven items assess anxiety symptoms. A 4‐point Likert scale was used, with individual item scores ranging from 0 to 3. Both the Anxiety subscale and Depression subscale scores range from 0 to 21, with higher scores indicating more severe anxiety or depressive symptoms. The present study obtained authorization from the original authors to use this scale, which has been fully validated for application in Chinese populations.

#### 2.7.5. Sleep Quality

Sleep quality over the preceding 1 month was assessed using the Pittsburgh Sleep Quality Index (PSQI) [35], a self‐report questionnaire employing a 4‐point Likert scale. Individual item scores range from 0 to 3, with total PSQI scores ranging from 0 to 21. Higher scores indicate poorer sleep quality. We acquired formal permission from the original authors for scale utilization, and this instrument has been validated among Chinese individuals.

#### 2.7.6. Physical Functional Capacity: 6MWD

6MWD was assessed as an objective measure of physical functional capacity. Participants were instructed to walk at the fastest pace they could sustain for 6 min. The test was administered by trained research personnel in a standardized manner.

#### 2.7.7. BMI

Body height and weight were measured using a professional electronic height and weight scale (Model: TCS‐200‐RT; Manufacturer: Wuxi Weighing Apparatus Factory Co., Ltd., China). All anthropometric measurements were conducted by trained research staff following standardized operating procedures to ensure accurate BMI calculation.

#### 2.7.8. Serum Creatinine

Reference values for serum creatinine were determined based on fasting blood samples collected on the day of the patient’s follow‐up visit.

### 2.8. Statistical Methods

Statistical analyses were performed using IBM SPSS Statistics Version 28.0. For continuous variables, the Shapiro–Wilk test was used to assess normality. Normally distributed data were expressed as mean ± standard deviation (mean ± SD), while non‐normally distributed data were presented as median and interquartile range (median [IQR]). Categorical variables were described using frequencies (*n*) and percentages (%).

For comparisons of continuous variables between the two groups, the independent samples *t*‐test was used for normally distributed data, and the Mann–Whitney *U* test was employed for non‐normally distributed data. To assess intragroup changes before and after the intervention, the paired samples *t*‐test was used if the differences followed a normal distribution; otherwise, the Wilcoxon signed‐rank test was applied. All statistical tests were two‐tailed, and a *p* value < 0.05 was considered statistically significant. Data analysis was conducted based on the intention‐to‐treat (ITT) principle, whereby all randomized participants were included in the analysis according to their original group assignment, regardless of intervention completion or loss to follow‐up. Missing values resulting from participant dropout were imputed using the K‐nearest neighbors (KNN) imputation method.

## 3. Results

### 3.1. Recruitment

From June to October 2025, a recruitment process was conducted at the renal transplant follow‐up outpatient clinic of a tertiary Grade A hospital in Jilin Province. A total of 103 patients were initially screened for eligibility. Of these, 39 patients were excluded, including 27 who did not meet the inclusion criteria and 12 who declined to participate. Consequently, 64 participants were enrolled and randomly assigned to either the intervention group (*n* = 32) or the control group (*n* = 32) in a 1:1 ratio. In the intervention group, 29 participants completed the Tai Chi exercise program, while 3 participants withdrew due to work commitments or lack of time. In the control group, 30 participants completed the final assessment, while 2 were lost to follow‐up and thus excluded from the final analysis (Figure 1).

**FIGURE 1:**
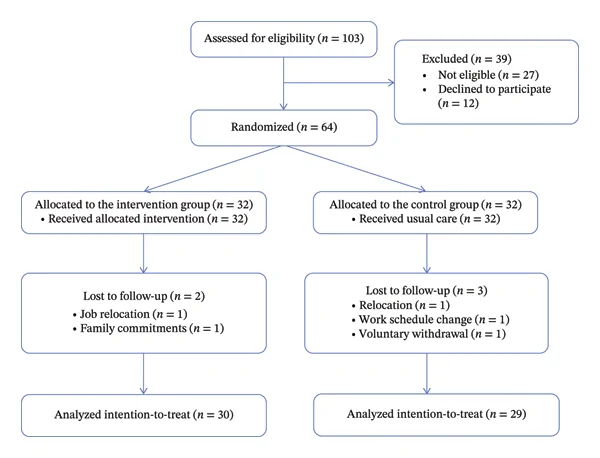
CONSORT flow diagram.

### 3.2. Baseline Characteristics

A total of 64 participants were enrolled in this study, of whom 31 (48.4%) were male. The median age was 50.0 years (IQR: 37.3, 53.0) in the control group and 42.5 years (IQR: 35.3, 51.8) in the experimental group. Regarding socioeconomic characteristics, 67.2% of the participants held a bachelor’s degree or higher, and 29.7% were unemployed. Detailed baseline demographic and clinical characteristics are presented in Table 2, while Table 3 summarizes the baseline values for all measured outcome indicators. Statistical analysis revealed no significant differences between the two groups across any baseline characteristics or outcome measures. No adverse events occurred in either group of participants.

**TABLE 2:** Participant demographic characteristics (*n* = 64).

Characteristic	Intervention group *n* (%)	Control group *n* (%)	Z/χ^2^	*p*
Sex				
Male	16 (50.0)	15 (46.9)	0.063^a^	0.802
Female	16 (50.0)	17 (53.1)		
Age (median, IQR, 25:75%)	42.5 (35.3,51.8)	50.0 (37.3,53.0)	−1.284^b^	0.199
Marital status				
Single/divorced/widowed	7 (21.9)	7 (21.9)	0.000^a^	1.000
Married	25 (78.1)	25 (78.1)		
Education level				
Master’s degree or above	1 (3.1)	1 (3.1)	—	0.673
Associate/bachelor’s degree	22 (68.8)	19 (59.4)		
Secondary education	5 (15.6)	7 (21.9)		
Primary education or below	4 (12.5)	5 (15.6)		
Employment				
Unemployed	7 (21.9)	12 (37.5)	—	0.467
Retired	10 (31.3)	6 (18.8)		
Worker	1 (3.1)	2 (6.2)		
Other occupations	14 (43.8)	12 (37.5)		
Household income (RMB)				
< 3000	9 (28.1)	10 (31.3)	—	0.088
3000–6000	9 (28.1)	13 (40.6)		
6000–10,000	6 (18.8)	8 (25.0)		
> 10,000	8 (25.0)	1 (3.1)		
Payment method				
Medical insurance	30 (93.8)	28 (87.5)	0.184^a^	0.668
Self‐pay	2 (6.2)	4 (12.5)		
Dialysis duration (years)				
> 5	7 (21.9)	6 (18.8)	—	0.493
3–5	7 (21.9)	3 (9.4)		
1–3	10 (31.3)	14 (43.8)		
< 1	8 (25.0)	9 (28.1)		
Postoperative complications				
Yes	3 (9.4)	5 (15.6)	0.143^a^	0.705
No	29 (90.6)	27 (84.4)		

*Note:* —, Indicates Fisher’s exact test was applied.

^a^Denotes the chi‐squared (χ^2^) test value.

^b^Denotes the *Z* test value.

**TABLE 3:** Baseline characteristics of all outcome measures (*n* = 64).

Outcomes	Intervention group (*n* = 32)	Control group (*n* = 32)	*t*/*Z*	*p*
*Fatigue*				
General fatigue, mean ± SD	10.3 ± 4.0	10.6 ± 2.9	−0.320^a^	0.750
Physical fatigue, mean ± SD	11.6 3 ± 3.9	11.7 ± 2.2	−0.157^a^	0.876
Mental fatigue, mean ± SD	9.3 ± 4.2	8.7 ± 3.4	0.621^a^	0.537
Reduced activity, mean ± SD	10.7 ± 3.0	11.8 ± 2.9	−1.465^a^	0.148
Reduced motivation, mean ± SD	8.6 ± 2.9	9.0 ± 2.5	−0.601^a^	0.550
Total fatigue score, mean ± SD	50.4 ± 15.2	51.8 ± 9.6	−0.412^a^	0.682

*Quality of life*				
Physical health, mean ± SD	48.9 ± 7.7	45.3 ± 7.4	1.909^a^	0.061
Mental health, mean ± SD	49.9 ± 10.4	50.5 ± 13.1	−0.195^a^	0.846

*Anxiety and depression*				
Anxiety, median, IQR, 25:75%	3.0 (1.0, 6.0)	5.0 (3.3, 7.0)	−1.822^b^	0.069
Depression, median, IQR, 25:75%	3.0 (1.3, 6.0)	2.0 (0.0, 4.0)	−1.560^b^	0.119
Sleep quality, median, IQR, 25:75%	7.0 (6.0, 8.0)	7.0 (5.3, 7.8)	−0.393^b^	0.694

*Six-min walk distance (m)*				
Mean ± SD	479.4 ± 58.4	467.6 ± 56.3	1.355^a^	0.180

*Clinical characteristics*				
BMI (kg/m^2^), mean ± SD	21.2 ± 2.4	21.1 ± 3.8	0.176^a^	0.861
Serum creatinine (μmol/L) median, IQR, 25:75%	103.1 (82.0, 136.5)	115.0 (96.5, 131.5)	−0.624^b^	0.532

*Note:* Physical health and mental health scores were standardized.

^a^Denotes the *t*‐value.

^b^Denotes the *Z*‐value.

### 3.3. Primary Outcome

The results demonstrated that after 12 weeks of Tai Chi intervention, there was a statistically significant difference in total fatigue scores between the two groups of renal transplant recipients (*p* < 0.05). The fatigue scores across all dimensions in the intervention group were significantly lower than those in the control group, with statistically significant differences (Table 4). Within‐group analysis revealed that the intervention group exhibited significant reductions in both total fatigue scores and all dimension subscores from baseline to 12 weeks postintervention (*p* < 0.05, Table 5). Conversely, the control group showed no significant changes in fatigue scores between baseline and the 12‐week follow‐up (*p* > 0.05, Table 6). In the sensitivity analysis using KNN imputation to handle missing data, the difference in fatigue scores between the intervention group and the control group remained statistically significant.

**TABLE 4:** Comparison of outcomes between the intervention and control groups based on complete case analyses (*n* = 59).

Outcomes	Intervention group (*n* = 30)	Control group (*n* = 29)	*t*/*Z*	*p*
*Fatigue*				
General fatigue, mean ± SD	7.9 ± 1.7	10.6 ± 2.5	−4.878^a^	< 0.001
Physical fatigue, mean ± SD	9.2 ± 2.3	11.5 ± 2.0	−4.152^a^	< 0.001
Mental fatigue, mean ± SD	7.2 ± 2.2	9.1 ± 3.3	−2.489^a^	0.016
Reduced activity, mean ± SD	9.7 ± 2.5	11.8 ± 2.5	−3.200^a^	0.002
Reduced motivation, mean ± SD	7.3 ± 2.0	9.0 ± 2.2	−2.971^a^	0.004
Total fatigue score, mean ± SD	41.3 ± 8.0	52.1 ± 8.6	−4.971^a^	< 0.001

*Quality of life*				
Physical health, mean ± SD	49.7 ± 8.1	45.9 ± 6.7	1.954^a^	0.056
Mental health, mean ± SD	52.4 ± 6.8	49.7 ± 12.0	1.045^a^	0.302

*Anxiety and depression*				
Anxiety, median, IQR, 25:75%	3.0 (3.0, 5.0)	6.0 (5.0, 7.5)	−3.941^b^	< 0.001
Depression, median, IQR, 25:75%	3.0 (1.0, 5.0)	3.0 (1.0, 5.0)	−0.536^b^	0.592
Sleep quality, median, IQR, 25:75%	6.0 (5.0, 7.0)	7.0 (6.0, 10.5)	−2.552^b^	0.011

*Six-min walk distance (m)*				
Mean ± SD	490.2 ± 50.7	457.9 ± 66.6	2.103^a^	0.040

*Clinical characteristics*				
BMI (kg/m^2^), mean ± SD	21.2 ± 1.8	21.2 ± 3.4	0.052^a^	0.959

*Serum creatinine (μmol/L)*				
M (P_25_, P_75_)	102.6 (68.8, 125.5)	116.0 (96.5, 132.0)	−1.319^b^	0.187

*Note:* Physical health and mental health scores were standardized.

^a^Denotes the *t*‐value.

^b^Denotes the *Z*‐value.

**TABLE 5:** Baseline to postintervention comparison within intervention group (*n* = 30).

Outcomes	Intervention group		*t*/*Z*	*p*
	Baseline (*n* = 30)	Postintervention (*n* = 30)		
General fatigue, mean ± SD	10.2 ± 4.1	7.9 ± 1.7	3.230^a^	0.003
Physical fatigue, mean ± SD	11.4 ± 4.0	9.2 ± 2.3	2.816^a^	0.009
Mental fatigue, mean ± SD	9.2 ± 4.1	7.2 ± 2.2	2.501^a^	0.018
Reduced activity, mean ± SD	11.1 ± 2.8	9.7 ± 2.5	2.191^a^	0.037
Reduced motivation, mean ± SD	8.7 ± 2.8	7.3 ± 2.0	2.099^a^	0.045
Total fatigue score, mean ± SD	50.2 ± 15.1	41.3 ± 8.0	3.155^a^	0.004
Physical health, mean ± SD	49.2 ± 7.8	49.7 ± 8.1	−0.266^a^	0.792
Mental health, mean ± SD	51.0 ± 6.8	52.4 ± 6.8	−0.637^a^	0.529
Anxiety, median, IQR, 25:75%	4.0 (2.0, 6.3)	3.0 (3.0, 5.0)	−0.697^b^	0.486
Depression, median, IQR, 25:75%	3.0 (1.0, 5.3)	3.0 (1.0, 5.0)	−1.006^b^	0.314
Sleep quality, median, IQR, 25:75%	7.0 (6.0, 9.3)	6.0 (5.0, 7.0)	−2.047^b^	0.041
Six‐min walk distance (m), mean ± SD	477.4 ± 59.9	490.2 ± 50.7	−2.385^a^	0.024
BMI (kg/m^2^), mean ± SD	21.0 ± 2.3	21.2 ± 1.8	−0.319^a^	0.752
Serum creatinine (μmol/L), *M* (P_25_, P_75_)	109.1 (86.0, 138.0)	102.6 (68.8, 125.5)	−0.541^b^	0.589

*Note:* Physical health and mental health scores were standardized.

^a^Denotes the *t*‐value.

^b^Denotes the *Z*‐value.

**TABLE 6:** Baseline to postintervention comparison within the control group (*n* = 29).

Outcomes	Control group		*t*/*Z*	*p*
	Baseline (*n* = 29)	Postintervention (*n* = 29)		
General fatigue, mean ± SD	10.7 ± 3.1	10.6 ± 2.5	0.090^a^	0.929
Physical fatigue, mean ± SD	11.8 ± 2.2	11.5 ± 2.0	0.573^a^	0.571
Mental fatigue, mean ± SD	8.8 ± 3.5	9.1 ± 3.3	−0.231^a^	0.819
Reduced activity, mean ± SD	11.9 ± 3.0	11.8 ± 2.5	0.135^a^	0.894
Reduced motivation, mean ± SD	9.1 ± 2.5	9.0 ± 2.2	0.340^a^	0.736
Total fatigue score, mean ± SD	52.3 ± 9.9	52.1 ± 8.6	0.103^a^	0.919
Physical health, mean ± SD	44.9 ± 7.2	45.9 ± 6.7	−0.643^a^	0.525
Mental health, mean ± SD	51.0 ± 6.8	49.7 ± 12.0	−0.217^a^	0.302
Anxiety, median, IQR, 25:75%	5.0 (3.0, 7.0)	6.0 (5.0, 7.5)	−0.243^b^	0.808
Depression, median, IQR, 25:75%	3.0 (0.5, 4.5)	3.0 (1.0, 5.0)	−0.565^b^	0.572
Sleep quality, median, IQR, 25:75%	7.0 (5.5, 9.5)	7.0 (6.0, 10.5)	−0.201^b^	0.841
Six‐min walk distance (m), mean ± SD	460.4 ± 70.3	457.9 ± 66.6	0.173^a^	0.864
BMI (kg/m^2^), mean ± SD	21.1 ± 4.0	21.2 ± 3.4	−0.139^a^	0.890
Serum creatinine (μmol/L), *M* (P_25_, P_75_)	120.0 (98.0, 132.5)	116.0 (96.5, 132.0)	−0.162^b^	0.871

*Note:* Physical health and mental health scores were standardized.

^a^Denotes the *t*‐value.

^b^Denotes the *Z*‐value.

### 3.4. Secondary Outcomes

Compared with the control group, the intervention group demonstrated significant improvements in anxiety (median: 3.0, IQR: 3.0–5.0), sleep quality (median: 6.0, IQR: 5.0–7.0), and 6MWD (mean: 490.2, SD: 50.7). However, no significant improvements were observed in quality of life, depression, BMI, and serum creatinine (*p* > 0.05, Table 4).

### 3.5. Sensitivity Analyses

After handling missing data using KNN imputation, the between‐group difference in fatigue remained statistically significant. The statistical significance of other key secondary outcomes, such as 6MWD and sleep quality, also remained unchanged. Of note, two measures showed changes in statistical inference in the sensitivity analysis. The “physical health” dimension of quality of life reached statistical significance after imputation (*p* = 0.043), whereas it demonstrated only a marginal trend in the primary analysis (*p* = 0.056). Conversely, anxiety scores showed highly significant between‐group differences in the primary analysis (*p* < 0.001), but this difference was no longer statistically significant after imputation (*p* = 0.102, Table A1).

## 4. Discussion

This is a 12‐week, three‐phase randomized controlled trial to investigate the effects of Tai Chi exercise on fatigue among kidney transplant recipients. The results demonstrated that the Tai Chi intervention significantly reduced fatigue scores, effectively alleviating fatigue symptoms in this population. Furthermore, improvements were observed in sleep quality, anxiety levels, and 6MWD. Notably, with the exception of a few participants who were unable to attend due to work, the majority of patients exhibited high adherence, suggesting a high level of acceptability for Tai Chi exercise among kidney transplant recipients.

### 4.1. Effect of the Tai Chi Exercise on Fatigue

Compared to the control group of renal transplant recipients who showed a tendency toward worsening fatigue after 12 weeks, regular Tai Chi exercise significantly improved the fatigue status of the intervention group, with significant improvements in both the total fatigue score and the scores of each dimension. This result is highly consistent with the findings of a previous randomized controlled trial in which Zhang et al. conducted a 3‐month Chinese traditional exercise, Baduanjin, combined with a resistance training program among renal transplant recipients; after the intervention, the fatigue scores of patients in the intervention group across five dimensions of the fatigue questionnaire were lower than those of patients in the control group [21]. In addition, the results of a systematic review also indicated that Tai Chi and Baduanjin can effectively alleviate fatigue in breast cancer patients [36].

The efficacy of Tai Chi in mitigating fatigue lies in its unique mechanism as a multidimensional mind–body exercise. The low‐to‐moderate intensity movements enhanced musculoskeletal strength [37] and aerobic capacity [38], addressing the physiological origins of exhaustion. Additionally, through the synergistic combination of deep breathing and slow, deliberate movements, Tai Chi may help attenuate stress levels and optimize autonomic nervous system function in transplant recipients [39], potentially contributing to fatigue amelioration. The substantial alleviation of fatigue observed in the intervention group may be further attributed to the individualized adaptability inherent in Tai Chi exercise. Tai Chi provides a low‐to‐moderate intensity, slow‐paced movement modality that permits flexible modifications according to each recipient’s physical capacity, thereby mitigating exercise‐related adverse effects and augmenting intervention adherence, which collectively ensures sustained amelioration of fatigue manifestations.

### 4.2. Effect of the Tai Chi Exercise on Secondary Outcomes

The intervention group demonstrated significantly superior sleep quality compared to the control group after 12 weeks of tai chi intervention, suggesting that tai chi exercise effectively improves sleep in kidney transplant recipients. In a randomized clinical trial [40], 29 kidney transplant recipients who engaged in regular exercise for 10 weeks exhibited a 27% improvement in sleep quality and a 30‐min increase in sleep duration. A meta‐analysis further demonstrated that tai chi concurrently improved both subjective and objective sleep quality measures, with sustained benefits observed during long‐term follow‐up. Notably, tai chi, yoga, and walking or jogging showed superior sleep‐improving efficacy compared to other exercise interventions [41]. The physiological mechanisms through which Tai Chi improves sleep quality may be mediated by multifaceted pathways. However, the underlying mechanisms remain largely unclear. These pathways include the enhancement of parasympathetic nervous system activity, modulation of heart rate variability, regulation of body temperature, and optimization of hormonal regulation [42, 43]. As a moderate‐intensity mind–body intervention that integrates physical movement, respiratory control, and psychological regulation, Tai Chi offers a holistic approach that appears more efficacious in enhancing sleep quality for kidney transplant recipients than isolated forms of exercise.

Furthermore, our findings demonstrated that Tai Chi exercise significantly increased the 6MWD among participants. This result was consistent with a previous randomized controlled trial, which reported that a 12‐week graded exercise program for renal transplant recipients—incorporating home‐based aerobic, resistance, and flexibility training modules—resulted in significantly better 6MWD performance in the experimental group compared to the control group [22]. The implementation of Tai Chi interventions in stroke patients had also been shown to effectively enhance motor function, further validating the advantages of Tai Chi in improving physical capacity [44]. This improvement may be attributed to the specific biomechanics of Tai Chi, where movements such as semisquats and weight shifts serve as lower‐limb training that strengthens muscular force and endurance, thereby directly enhancing walking ability. Additionally, Tai Chi’s emphasis on physical coordination and equilibrium contributes to greater gait stability and efficiency [37].

In contrast, no significant improvements in BMI or serum creatinine levels were observed in this study, which aligns with the findings of a previous study [21] and a systematic review [45]. Several factors may explain these results. First, renal function is influenced by a complex array of physiological variables, and exercise intervention as a standalone modality may have limited impact on biochemical markers like creatinine. Second, as a moderate‐intensity mind–body exercise, the energy expenditure associated with Tai Chi is relatively low, which may be insufficient to induce significant changes in BMI. Furthermore, the participants in this study maintained relatively good baseline renal function, which inherently restricted the margin for measurable physiological improvement. Nevertheless, these limitations ensured the homogeneity and safety of the study cohort. Importantly, the absence of any adverse events during the intervention period confirms the safety and feasibility of Tai Chi for renal transplant recipients.

Regarding psychological indicators, no statistically significant improvements were observed in depression scores or the “mental health” dimension of quality of life. This finding contradicts previous research. A Baduanjin intervention trial for kidney transplant recipients reported a significant reduction in depressive symptoms [21]. The discrepancy may stem from the fact that the intervention group in that study utilized multimedia tools—such as rehabilitation videos—to reinforce learning and enhance self‐management capabilities. The etiology of depression is complex, involving multifaceted interactions across biological, psychological, and sociological dimensions [46]. While isolated mind–body exercise interventions can yield positive effects, managing the multifactorial nature of depressive states may require integrated strategies that combine exercise with cognitive‐behavioral therapy or psychological counseling to achieve significant clinical outcomes.

Intriguingly, the sensitivity analysis revealed fluctuations in several statistical inferences following the application of specific missing data handling techniques. Specifically, while the reduction in anxiety scores demonstrated statistical significance in the primary analysis, this effect was attenuated and became nonsignificant after KNN imputation. Conversely, the “physical health” domain of the quality‐of‐life assessment reached statistical significance only after the imputation process. On balance, the core findings of this study—that Tai Chi intervention significantly enhances exercise capacity and alleviates fatigue—remained robust and insensitive to the methods used for handling missing data. However, the evidence regarding the intervention’s impact on anxiety and “physical health” domains exhibited less stability, suggesting that these secondary outcomes should be further validated in larger‐scale trials.

### 4.3. Limitations

Despite the valuable insights provided by this study, several limitations must be acknowledged. First, this study was conducted only in a tertiary hospital in Changchun, and the single source of samples may limit the generalizability of the results. Future research should prioritize multicenter randomized controlled trials with larger, more diverse cohorts to enhance the external validity of the results. Second, the duration of the intervention was limited to 12 weeks, and the absence of a long‐term follow‐up period precludes any conclusions regarding the sustained effects of the program. Subsequent studies should incorporate extended longitudinal monitoring to evaluate the durability of the intervention outcomes over time. Finally, this study did not stratify participants based on the time elapsed since transplantation. Given that the physiological and psychological challenges may vary significantly between the early postoperative phase and the long‐term recovery period, future investigations should conduct subgroup analyses based on transplantation vintage to provide more tailored and precise clinical recommendations.

### 4.4. Implications for Clinical Practice

For kidney transplant recipients, engaging in physical exercise presents not only physiological challenges but also substantial psychological barriers. Many patients decline participation in exercise due to concerns that physical activity may compromise the function of the transplanted kidney [25]. As a low‐intensity exercise modality characterized by gentle, flowing movements, Tai Chi offers enhanced safety profiles and is therefore more likely to be accepted by kidney transplant recipients. A previous 12‐month remote exercise intervention study demonstrated improvements in quality of life and cardiopulmonary fitness among transplant recipients; however, only 65% of participants completed the entire intervention period through final assessment [47]. In contrast, this study achieved an overall dropout rate of only 7.8%, indicating that participants demonstrated high acceptance and good adherence to the Tai Chi intervention. This suggests that such an intervention program may be more efficacious in enabling kidney transplant recipients to derive sustainable benefits from exercise.

## 5. Conclusions

This study demonstrated that a 12‐week Tai Chi intervention significantly improved the primary outcome of fatigue among renal transplant recipients, with additional secondary benefits observed in sleep quality and exercise capacity. These results suggest that Tai Chi, characterized by its low‐impact nature and integration of mind–body coordination, may represent a viable and effective nonpharmacological adjunct for symptom management.

## Funding

No funding was received for this manuscript.

## Conflicts of Interest

The authors declare no conflicts of interest.

## Data Availability

Data will be available on request from the authors.

## Appendix 1

**Table A1:** Intention‐to‐treat analysis of missing values in outcome measures.

Outcomes	Intervention group (*n* = 32)	Control group (*n* = 32)	*t/Z*	*p*
*Fatigue*				
General fatigue, Mean ± SD	8.09 ± 1.89	10.84 ± 2.69	−4.733^a^	< 0.001
Physical fatigue, Mean ± SD	9.47 ± 2.51	11.69 ± 2.04	−3.877^a^	< 0.001
Mental fatigue, Mean ± SD	7.53 ± 2.46	9.31 ± 3.26	−2.468^a^	0.016
Reduced activity, Mean ± SD	10.00 ± 2.64	11.97 ± 2.44	−3.097^a^	0.003
Reduced motivation, Mean ± SD	7.38 ± 1.95	9.00 ± 2.13	−3.189^a^	0.002
Total fatigue score, Mean ± SD	42.34 ± 8.75	53.00 ± 8.69	−4.888^a^	< 0.001

*Quality of life*				
Physical health, Mean ± SD	49.74 ± 7.88	46.04 ± 6.35	2.069^a^	0.043
Mental health, Mean ± SD	52.61 ± 6.71	50.31 ± 11.60	0.971^a^	0.336

*Anxiety and Depression*				
Anxiety, median, IQR, 25:75%	3.50 (3.00, 5.25)	6.00 (3.00, 7.00)	−1.634^b^	0.102
Depression, median, IQR, 25:75%	3.00 (1.00, 5.00)	3.00 (1.75, 5.00)	−0.258^b^	0.797
Sleep quality , median, IQR, 25:75%	6.50 (5.75, 7.00)	7.50 (6.00, 9.25)	−2.625^b^	0.009
Six‐minute walk distance (m) Mean ± SD	489.36 ± 49.15	457.73 ± 63.57	2.227^a^	0.030

*Clinical characteristics*				
BMI (kg/m^2^) Mean ± SD	21.18 ± 1.80	21.02 ± 3.28	0.236^a^	0.815
Serum creatinine (μmol/L) *M* (P_25_, P_75_)	103.55 (90.25, 127.50)	117.20 (98.50, 127.05)	−1.142^b^	0.254

*Note:* Physical health and mental health scores were standardized.

^a^Denotes the *t*‐value.

^b^Denotes the *Z*‐value.

## References

[1] Jager K. J. , Kovesdy C. , Langham R. , Rosenberg M. , Jha V. , and Zoccali C. , A Single Number for Advocacy and Communication-Worldwide More Than 850 Million Individuals Have Kidney Diseases, Kidney International. (2019) 96, no. 5, 1048–1050, 10.1016/j.kint.2019.07.012. 31582227

[2] Liyanage T. , Toyama T. , Hockham C. et al., Prevalence of Chronic Kidney Disease in Asia: A Systematic Review and Analysis, BMJ Global Health. (2022) 7, no. 1, 10.1136/bmjgh-2021-007525. PMC8796212 35078812

[3] Nyokabi P. , Youngkong S. , Bagepally B. S. et al., A Systematic Review and Quality Assessment of Economic Evaluations of Kidney Replacement Therapies in End-Stage Kidney Disease, Scientific Reports. (2024) 14, no. 1, 10.1038/s41598-024-73735-8. PMC11450173 39362958

[4] Poggio E. D. , Augustine J. J. , Arrigain S. , Brennan D. C. , and Schold J. D. , Long-Term Kidney Transplant Graft Survival-Making Progress When Most Needed, American Journal of Transplantation. (2021) 21, no. 8, 2824–2832, 10.1111/ajt.16463. 33346917

[5] Tsarpali V. , Midtvedt K. , Lønning K. et al., Health-Related Quality of Life in Older Kidney Transplant Recipients: A National Cohort Study of Short- and Longer-Term Outcomes, Kidney Med. (2021) 3, no. 6, 974–983, 10.1016/j.xkme.2021.05.007. PMC8664696 34939006

[6] Sands I. , Picariello F. , Maple H. , and Chilcot J. , Psychosocial and Clinical Associations of Fatigue Severity and Fatigue-Related Impairment in Kidney Transplant Recipients, Behavioral Medicine. (2024) 50, no. 2, 153–163, 10.1080/08964289.2022.2159311. 36803685

[7] King C. P. , Cossart A. R. , Isbel N. M. , Campbell S. B. , and Staatz C. E. , The Association Between Tacrolimus Exposure and Tremor, Headache and Insomnia in Adult Kidney Transplant Recipients: A Systematic Review, Transplantation Reviews. (2024) 38, no. 1, 10.1016/j.trre.2023.100815. 38071930

[8] Uyar B. , The Analysis of Immunosuppressant Therapy Adherence, Depression, Anxiety, and Stress in Kidney Transplant Recipients in the Post-Transplantation Period, Transplant Immunology. (2022) 75, 10.1016/j.trim.2022.101686. 35961441

[9] Malyszko J. , Golenia A. , Farisco M. et al., Cognitive Impairment in Kidney Transplanted Patients, Nephrology Dialysis Transplantation. (2025) 40, no. Supplement_2, ii46–ii53, 10.1093/ndt/gfae250. PMC11905761 40080090

[10] Bossola M. , Arena M. , Urciuolo F. et al., Fatigue in Kidney Transplantation: A Systematic Review and Meta-Analysis, Diagnostics. (2021) 11, no. 5, 10.3390/diagnostics11050833. PMC8147914 34063041

[11] Liu S. , Shen Y. , Nie M. et al., The Status and Influencing Factors of Fatigue in Kidney Transplant Recipients Based on the Theory of Unpleasant Symptoms: A Cross-Sectional Study in China, International Journal of Nursing Practice. (2024) 30, no. 5, 10.1111/ijn.13256. 38570821

[12] Chu A. Q. , Zhang H. L. , Chen J. , and Guan X. H. , Symptom Cluster and Influencing Factors in Kidney Transplant Patients at Home, Journal of Nursing Science. (2020) 35, 19–23.

[13] Knobbe T. J. , Kremer D. , Eisenga M. F. et al., Sleep Quality, Fatigue, Societal Participation and Health-Related Quality of Life in Kidney Transplant Recipients: A Cross-Sectional and Longitudinal Cohort Study, Nephrology Dialysis Transplantation. (2023) 39, no. 1, 74–83, 10.1093/ndt/gfad148. PMC10730797 37418245

[14] Liu M. , Xie J. , Sun Q. et al., Correlation of Objective and Subjective Sleep Parameters With Fatigue and Depression in Kidney Transplant Recipients, Zhong Nan Da Xue Xue Bao Yi Xue Ban. (2024) 49, 1279–1289, 10.11817/j.issn.1672-7347.2024.240157. PMC11628229 39788516

[15] Lee S. Y. , Chu S. H. , Oh E. G. , and Huh K. H. , Low Adherence to Immunosuppressants is Associated With Symptom Experience Among Kidney Transplant Recipients, Transplantation Proceedings. (2015) 47, no. 9, 2707–2711, 10.1016/j.transproceed.2015.09.056. 26680077

[16] Belaiche S. , DéCAUDIN B. , Dharancy S. , Noel C. , Odou P. , and Hazzan M. , Factors Relevant to Medication Non-Adherence in Kidney Transplant: A Systematic Review, International Journal of Clinical Pharmacy. (2017) 39, no. 3, 582–593, 10.1007/s11096-017-0436-4. 28374343

[17] Chen X. , Li J. , Chen C. et al., Effects of Exercise Interventions on Cancer-Related Fatigue and Quality of Life Among Cancer Patients: A Meta-Analysis, BMC Nursing. (2023) 22, no. 1, 10.1186/s12912-023-01363-0. PMC10261838 37312185

[18] Buğday B. , Çelik A. L. , Safran E. E. , and Şevgin Ö. , Impact of Resistance Exercise and Diet on Physical Activity, Sleep, and Fatigue in Obese Individuals: A Randomized Controlled Trial, BMC Public Health. (2025) 25, no. 1, 10.1186/s12889-025-23549-8. PMC12220626 40604657

[19] Grigoriou S. S. , Krase A. A. , Karatzaferi C. et al., Long-Term Intradialytic Hybrid Exercise Training on Fatigue Symptoms in Patients Receiving Hemodialysis Therapy, International Urology and Nephrology. (2021) 53, no. 4, 771–784, 10.1007/s11255-020-02711-8. 33387217

[20] Knobbe T. J. , Kremer D. , van der Meulen M. L. et al., Effect of Exercise or Combined Exercise and Diet Intervention on Sleep and Fatigue in Kidney Transplant Recipients, Kidney International Reports. (2025) 10, no. 7, 2233–2242, 10.1016/j.ekir.2025.04.008. PMC12266161 40677336

[21] Zhang P. , Liu S. , Zhu X. et al., The Effects of a Physical Exercise Program in Chinese Kidney Transplant Recipients: A Prospective Randomised Controlled Trial, Clinical Kidney Journal. (2023) 16, no. 8, 1316–1329, 10.1093/ckj/sfad065. PMC10387397 37529646

[22] Senthil Kumar T. G. , Soundararajan P. , Maiya A. G. , and Ravi A. , Effects of Graded Exercise Training on Functional Capacity, Muscle Strength, and Fatigue After Renal Transplantation: A Randomized Controlled Trial, Saudi Journal of Kidney Diseases and Transplantation. (2020) 31, no. 1, 100–108, 10.4103/1319-2442.279929. 32129202

[23] Torregrosa J. V. , Ferreira A. C. , Cucchiari D. , and Ferreira A. , Bone Mineral Disease After Kidney Transplantation, Calcified Tissue International. (2021) 108, no. 4, 551–560, 10.1007/s00223-021-00837-0. 33765230

[24] Sertorio E. S. , Colugnati F. A. B. , Denhaerynck K. et al., Factors Associated With Physical Inactivity of Recipients of a Kidney Transplant: Results From the ADHERE BRAZIL Multicenter Study, Physical Therapy. (July 2024) 104, no. 7, 10.1093/ptj/pzae058. 38591795

[25] Bates A. , Letton M. E. , Arnold R. , and Lambert K. , Barriers and Enablers to Exercise in Kidney Transplant Recipients: Systematic Review of Qualitative Studies, Journal of Renal Care. (2024) 50, no. 4, 384–404, 10.1111/jorc.12497. 38806247

[26] Liu M. , Sun Q. , Cui L. et al., Fear of Movement and Physical Self-Efficacy Partially Mediate the Association Between Fatigue and Physical Activity Among Kidney Transplant Recipients, Clinical Nursing Research. (2021) 30, no. 7, 950–959, 10.1177/1054773821990263. 33511857

[27] Cheng D. , Wang X. , Hu J. et al., Effect of Tai Chi and Resistance Training on Cancer-Related Fatigue and Quality of Life in Middle-Aged and Elderly Cancer Patients, Chinese Journal of Integrative Medicine. (2021) 27, no. 4, 265–272, 10.1007/s11655-021-3278-9. 33420583

[28] Wu K. , Li Y. , Zou Y. et al., Tai Chi Increases Functional Connectivity and Decreases Chronic Fatigue Syndrome: A Pilot Intervention Study With Machine Learning and FMRI Analysis, PLoS One. (2022) 17, no. 12, 10.1371/journal.pone.0278415. PMC9714925 36454926

[29] Yao L. Q. , Kwok S. W. H. , Tan J. B. et al., The Effect of an Evidence-Based Tai Chi Intervention on the Fatigue-Sleep Disturbance-Depression Symptom Cluster in Breast Cancer Patients: A Preliminary Randomised Controlled Trial, European Journal of Oncology Nursing. (2022) 61, 10.1016/j.ejon.2022.102202. 36228406

[30] Chang J. H. , Koo M. , Wu S. W. , and Chen C. Y. , Effects of a 12-Week Program of Tai Chi Exercise on the Kidney Disease Quality of Life and Physical Functioning of Patients With End-Stage Renal Disease on Hemodialysis, Complementary Therapies in Medicine. (2017) 30, 79–83, 10.1016/j.ctim.2016.12.002. 28137531

[31] Huang J. , Wang D. , and Wang J. , Clinical Evidence of Tai Chi Exercise Prescriptions: A Systematic Review, Evidence-Based Complementary and Alternative Medicine. (2021) 2021, 10.1155/2021/5558805.

[32] Han Q. F. and Tian J. , Reliability and Validity Verification of the Multidimensional Fatigue Inventory in Cancer Patients, Chinese Journal of Nursing. (2012) 47, no. 6, 548–550.

[33] Wang H. L. , Sinicization Study of the SF-36 Health Survey Scale, 2000, Zhejiang University.

[34] Sun Z. X. , Liu H. X. , Jiao L. Y. , Zhou T. , Yang L. N. , and Fan J. Y. , Reliability and Validity Study of the Hospital Anxiety and Depression Scale, Chinese Journal of Clinical Physicians (Electronic Edition). (2017) 11, 198–201.

[35] Lu T. Y. , Li Y. , Xia P. , Zhang G. Q. , and Wu D. R. , Reliability and Validity Analysis of the Pittsburgh Sleep Quality Index, Chongqing Medicine. (2014) 43, 260–263.

[36] Chen Y. , Zuo X. , Tang Y. , and Zhou Z. , The Effects of Tai Chi and Baduanjin on Breast Cancer Patients: Systematic Review and Meta-Analysis of Randomized Controlled Trials, Frontiers Oncology. (2024) 14, 10.3389/fonc.2024.1434087. PMC11551136 39529823

[37] Wehner C. , Blank C. , Arvandi M. , Wehner C. , and Schobersberger W. , Effect of Tai Chi on Muscle Strength, Physical Endurance, Postural Balance and Flexibility: A Systematic Review and Meta-Analysis, BMJ Open Sport Exerc Med. (2021) 7, no. 1, 10.1136/bmjsem-2020-000817. PMC7871341 33614126

[38] Tan T. , Meng Y. , Lyu J. L. et al., A Systematic Review and Meta-Analysis of Tai Chi Training in Cardiorespiratory Fitness of Elderly People, Evidence-Based Complementary and Alternative Medicine. (2022) 2022, 10.1155/2022/4041612.

[39] Duan D. , Huang H. , Li W. , Xiao C. , and Wang D. , Acute Effects of Tai Chi With Different Practice Emphasis on Autonomic Activity, Frontiers in Sports and Active Living. (2025) 7, 10.3389/fspor.2025.1642123. PMC12434074 40959532

[40] Pooranfar S. , Shakoor E. , Shafahi M. et al., The Effect of Exercise Training on Quality and Quantity of Sleep and Lipid Profile in Renal Transplant Patients: A Randomized Clinical Trial, International Journal of Organ Transplantation Medicine. (2014) 5, no. 4, 157–165. PMC4243047 25426284

[41] Bu Z. J. , Liu F. S. , Shahjalal M. et al., Effects of Various Exercise Interventions in Insomnia Patients: A Systematic Review and Network Meta-Analysis, BMJ Evid Based Med. (2026) 31, no. 2, 102–113, 10.1136/bmjebm-2024-113512. 40664502

[42] Irwin M. R. , Olmstead R. , and Motivala S. J. , Improving Sleep Quality in Older Adults With Moderate Sleep Complaints: A Randomized Controlled Trial of Tai Chi Chih, Sleep. (2008) 31, 1001–1008. PMC2491506 18652095

[43] Yu A. P. , Tam B. T. , Lai C. W. et al., Revealing the Neural Mechanisms Underlying the Beneficial Effects of Tai Chi: A Neuroimaging Perspective, American Journal of Chinese Medicine. (2018) 46, no. 02, 231–259, 10.1142/S0192415X18500131. 29542330

[44] Shao Y. , Han J. Y. , Li H. L. , Ren Z. P. , and Yang H. , Effect of Traditional Chinese Exercises on the Physical and Mental Health of Stroke Patients: A Meta-Analysis, Frontiers in Neurology. (2024) 15, 10.3389/fneur.2024.1455679. PMC11525336 39484051

[45] Billany R. E. , Bishop N. C. , Castle E. M. et al., Physical Activity Interventions in Adult Kidney Transplant Recipients: An Updated Systematic Review and Meta-Analysis of Randomized Controlled Trials, Renal Failure. (2025) 47, no. 1, 10.1080/0886022X.2025.2480246. PMC11951324 40148080

[46] Katon W. J. , Epidemiology and Treatment of Depression in Patients With Chronic Medical Illness, Dialogues in Clinical Neuroscience. (2011) 13, 7–23, 10.31887/DCNS.2011.13.1/wkaton. PMC3181964 21485743

[47] Vecchiato M. , Duregon F. , Zanardo E. et al., Tailored Exercise With Telehealth Monitoring Improves Adherence and Global Health in Kidney Transplant Recipients, Frontiers in Sports and Active Living. (2024) 6, 10.3389/fspor.2024.1436742. PMC11438482 39346494

